# Hybridization of Polymer-Encapsulated MoS_2_-ZnO Nanostructures as Organic–Inorganic Polymer Films for Sonocatalytic-Induced Dye Degradation

**DOI:** 10.3390/polym16152213

**Published:** 2024-08-02

**Authors:** Gowthami Palanisamy, Mrunal Bhosale, Sahil S. Magdum, Sadhasivam Thangarasu, Tae-Hwan Oh

**Affiliations:** School of Chemical Engineering, Yeungnam University, Gyeongsan 38541, Republic of Korea; mrunal.snst.1@gmail.com (M.B.); sai.nanoworld@gmail.com (S.S.M.); sadhasivam.nano@gmail.com (S.T.)

**Keywords:** hybrid film, PVDF, sonocatalyst, sonoluminescence, rhodamine B, dye degradation, organic pollutant removal, wastewater treatment, MoS_2_

## Abstract

The development of environmentally friendly technology is vital to effectively address the issues related to environmental deterioration. This work integrates ZnO-decorated MoS_2_ (MZ) to create a high-performing PVDF-based PVDF/MoS_2_-ZnO (PMZ) hybrid polymer composite film for sonocatalytic organic pollutant degradation. An efficient synergistic combination of MZ was identified by altering the ratio, and its influence on PVDF was assessed using diverse structural, morphological, and sonocatalytic performances. The PMZ film demonstrated very effective sonocatalytic characteristics by degrading rhodamine B (RhB) dye with a degradation efficiency of 97.23%, whereas PVDF only degraded 17.7%. Combining MoS_2_ and ZnO reduces electron–hole recombination and increases the sonocatalytic degradation performance. Moreover, an ideal piezoelectric PVDF polymer with MZ enhances polarization to improve redox processes and dye degradation, ultimately increasing the degradation efficiency. The degradation efficiency of RhB was seen to decrease while employing isopropanol (IPA) and p-benzoquinone (BQ) due to the presence of reactive oxygen species. This suggests that the active species •O_2_^−^ and •OH are primarily responsible for the degradation of RhB utilizing PMZ2 film. The PMZ film exhibited improved reusability without substantially decreasing its catalytic activity. The superior embellishment of ZnO onto MoS_2_ and effective integration of MZ into the PVDF polymer film results in improved degrading performance.

## 1. Introduction

Water pollution caused by organic pollutants has become a significant global concern because of the rapid increase in industry, particularly the release of polluted waste from the various processes of dyeing industries [1,2,3,4,5]. Various modernized methods have been developed to properly remediate polluted water [6,7,8,9]. Sonocatalysis has been a prominent method in recent times for efficiently breaking down many types of dyes among the several modern oxidation procedures [10,11]. Sonocatalysis-based approaches are very attractive for environmentally friendly dye degradation due to the broad and omnipresent nature of ultrasonic vibrations in all aquatic conditions [12,13]. Sonocatalytic degradation is able to break down organic contaminants because of the very reactive oxidizing agents, which are produced when microbubbles are rapidly formed, grown, and then collapsed [13]. A rapid buildup of high localized pressure (~1000 atm) and temperatures (~5000 K) probably occurs during the ultra-sonication process, which can generate oxidizing active radicals (like •H, •OH, •HO_2_) via sonoluminescence behavior by the emission of light [14,15,16,17,18].

Selecting reactive and high-performing catalyst materials is essential for efficient sonocatalytic dye degradation. Generally, the ultrasonic process energizes the catalyst and facilitates the generation of electrons (e^−^) from the valence band to the conduction band. This process results in the creation of energy and leaves behind holes (h^+^) [13]. In this connection, molybdenum disulfide (MoS_2_) has been verified as an efficient catalyst because of its intrinsic properties, mainly its ability to produce free radicals by generating electron–hole (*e–h*) pairs during the ultrasonic process [19,20]. The recombination of electron–hole pairs on the bulk MoS_2_ surface and the development of multilayers by restacking are specific limitations that have a substantial impact on the overall performance of sonocatalysis dye degradation [12,21,22]. Therefore, several attempts have been made to improve the overall degradation of dyes by the use of MoS_2_-based nanocomposites with different nanostructures as composite sonocatalysts and MoS_2_-containing polymer films [13,14,23]. The critical approaches primarily relate to (i) enhancing the performance by generating the synergy via altering the surface properties and (ii) preventing undesirable aggregation. Related to this, we examined the aforementioned notions in the present study.

The integration of zinc oxide (ZnO) nanoparticles onto the surface and between the layers of MoS_2_ can probably influence the overall performances of MoS_2_ in different scenarios for various applications [24,25,26,27]. ZnO has previously been shown to be an effective photocatalyst and sonocatalyst material for the degradation of different dyes, including rhodamine B (RhB) [28,29,30,31]. By incorporating ZnO with other semiconductor materials, it is possible to create heterogeneous catalysts that promote the synergistic effect of enhancing the separation of electron–hole pairs [32]. This phenomenon contributes to the production of a more significant number of reactive radicals together with hot spot theory and the sonoluminescence process [33,34]. In another way, incorporating catalyst material into the polymer matrix effectively prevents the aggregation of catalyst materials and improves pollutant degradation performance during extended periods of operation. Interest in polyvinylidene fluoride (PVDF)-based films for organic pollution removal has increased due to their piezoelectricity, adsorption capacity, stability, and inexpensive cost [23,35,36,37,38,39].

In this work, we developed a novel ZnO-decorated MoS_2_ in the PVDF (PMZ) film as a hybridized composite to achieve effective RhB degradation via sonocatalytic dye degradation. Different concentrations of MoS_2_ and ZnO (MZ) nanocomposite were developed to achieve efficient pollutant degradation performance. The efficient degradation of RhB was obtained from the homogeneous incorporation of MZ in the PVDF film compared to the PVDF-only film. The superior embellishment of ZnO on MoS_2_ and the successful integration of MZ in the PVDF polymer film led to enhanced degradation performance. As a result of the establishment of synergy, the PMZ composite structure effectively alters the individual activities of MoS_2_, ZnO, and PVDF towards degrading the RhB.

## 2. Materials and Methods

### 2.1. Materials

Polyvinylidene fluoride (PVDF, Mw ~534,000 by GPC) was procured from Sigma Aldrich, USA. Zinc acetate dihydrate (Zn(CH_3_COO)_2_·2H_2_O), sodium hydroxide (NaOH), thiourea and N,N-Dimethyl acetamide (DMAc) were obtained from DUKSAN reagent, Republic of Korea. Ammonium heptamolybate tetrahydrate ((NH_4_)_6_Mo_7_O_24_·4H_2_O, 99.0%) from Fischer chemical, UK. Rhodamine B (RhB), ethanol (C_2_H_5_OH, 94.5%), isopropanol (IPA, 99.5%) and p-benzoquinone (BQ) (C_6_H_4_O_2_) were acquired from Daejung chemicals & metals, Republic of Korea. Ethylenediamine tetraacetic acid (EDTA) (C_10_H_16_N_2_O_8_, 99%) was purchased from Acros Organics, Belgium. DI-water used in the present investigations.

### 2.2. Preparation of MoS_2_

MoS_2_ was synthesized by facile hydrothermal method [40]. At first, a desired amount of (NH_4_)_6_Mo_7_O_24_·4H_2_O was thermally treated in atmospheric air at 450 °C for 2 h to obtain molybdenum trioxide (MoO_3_). Subsequently, the desired amount of 0.23 g of as-prepared MoO_3_ and 0.5328 g of thiourea were added in 70 mL of DI water and stirred for 30 min. Afterwards, the reaction mixture was kept in a Teflon-lined autoclave and placed in a furnace at 200 °C for 24 h. After the reactor was cooled to room temperature, the black precipitate was carefully washed several times using ethanol and DI water. Finally, the product undergoes a drying process at a temperature of 60 °C.

### 2.3. Preparation of MoS_2_-ZnO Nanocomposite

**Figure 1:**
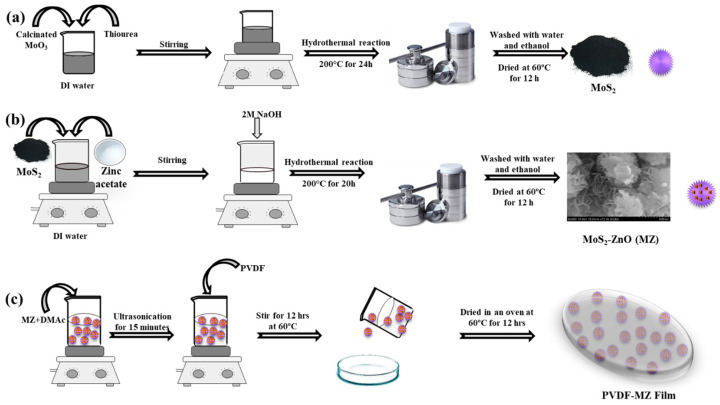
Schematic illustrations of (**a**) MoS_2_, (**b**) MoS_2_-ZnO nanocomposite, and (**c**) PVDF–MZ hybrid composite film preparation steps.

Different weight ratios of MoS_2_ and Zn(CH_3_COO)_2_·2H_2_O (9.5:0.5, 9:1, 8:2) were used to develop the MoS_2_-ZnO nanocomposite to estimate the most efficient catalyst material among the various combinations, and the preparation process of MoS_2_-ZnO is schematically illustrated in Figure 1b. Following the dispersion of 0.475 g MoS_2_ in 52.5 mL of DI water, the 0.025 g Zn(CH_3_COO)_2_·2H_2_O was added to the solution under continuous stirring. After adding Zn(CH_3_COO)_2_·2H_2_O, the solution was stirred for 10 min. Following that, the pH of the solution was adjusted to pH 12 through the addition of 2 M NaOH. The reaction mixture was then transferred to an autoclave lined with Teflon and heated to 200 °C for 20 h. To attain the synthesis of the nanocomposite, the previously mentioned centrifugation–redispersion process was employed several times. Then, the final products (MoS_2_-ZnO) of the different concentrations of MoS_2_ and ZnO (9.5:0.5, 9:1, and 8:2) were denoted as MZ1, MZ2, and MZ3 and utilized after being dried in a vacuum oven.

### 2.4. Fabrication of Hybrid PVDF–MZ Composite Film

As represented in Figure 1c, the PVDF film with and without MoS_2_ and MZ was prepared. For preparing the hybrid composite film, the 2 wt% of as-prepared MZ nanocomposite powder (MZ1, MZ2, and MZ3) was distributed in DMAc solution using ultrasonic vibration for attaining complete dispersion. Afterward, 0.5 g of PVDF was added into the above-mentioned solution and kept for magnetic stirring at 60 °C for 12 h. After completing the reaction, the solution was cast on a petri dish and dried in an oven at 60 °C. The reinforcement of different ratios of MZ1, MZ2, and MZ3 in the PVDF is denoted as PMZ1, PMZ2, and PMZ3, respectively. The pristine PVDF film was prepared without MoS_2_ and MZ nanocomposite powders following the same procedure. Meanwhile, PVDF–MoS_2_ films (PM) are prepared using the same procedure with the addition of as-prepared MoS_2_ powder.

### 2.5. Characterization Techniques

XRD diffraction analysis was performed by X-ray diffractometer (PANalytical X’pert, Malvern, UK). The measurements were performed within 10 and 80 degrees of diffraction angle (2θ), with a scan step size of 0.02° used for the analysis. The functional groups of the prepared film were analyzed using Fourier transform infrared spectroscopy (FT-IR spectroscopy, Perkin Elmer, Spectrum 100, USA). Additionally, X-ray photoelectron spectroscopy (XPS) (Thermo Fisher Scientific K-α surface analysis) was employed to verify the composition of the composite material. The surface morphologies and elemental composition of the as-prepared materials and films were investigated through a field-emission scanning electron microscope (FE-SEM) (Hitachi S-4800 with EDAX, Japan). Using transmission electron microscopy (TEM, Hitachi H-7600, Japan), the dimensions and morphology of the as-prepared catalysts were analyzed. The sonocatalytic degradation measurements were examined through UV/vis spectrophotometer (Ubi-600, MicroDigital Co., Ltd., Republic of Korea)) with a wavelength range of 400–700 nm.

### 2.6. Sonocatalytic Experiment

The sonocatalytic efficiency of the as-prepared composite film was investigated by degrading the RhB-containing solution in the ultrasonic bath, which maintained a temperature of 25 ± 3 °C. In a typical experiment, the film was immersed in 100 mL of 10 ppm RhB solution. To achieve an adsorption–desorption equilibrium between the RhB and the catalyst, the RhB solution was subsequently stirred for 30 min in the dark through a magnetic agitating apparatus. Afterwards, the reaction was carried out in an ultrasonic bath (Powersonic 620, Kleentek, Australia) for 75 min to induce ultrasonic vibration with a frequency of 50 kHz with 250 W power dissipation. At regular time intervals (15 min), a desired sample quantity was collected, and the absorbance was measured using a UV–vis spectrophotometer. All the experiments were performed at room temperature. The degradation efficiency was calculated using the following equation:

Degradation efficiency (%) = (C_0_ − C)/C_0_ × 100 (1)

Here, $C_{0}$ and C represent the initial dye concentration and concentration of dyes with different reaction times, respectively. The sonocatalytic degradation mechanism was elucidated by quantifying the generation of reactive oxygen species (ROS) by incorporating 10 mM of scavengers (EDTA, IPA, and BQ) into the aqueous solution. Additionally, the stability and reusability of the composite film were assessed using cyclic performances. After each successive cycle, the used film was removed and washed with DI water to eliminate the dyes on the film. Subsequently, the film was subjected to a drying process at a temperature of 60 °C in preparation for further studies.

## 3. Results and Discussion

**Figure 2:**
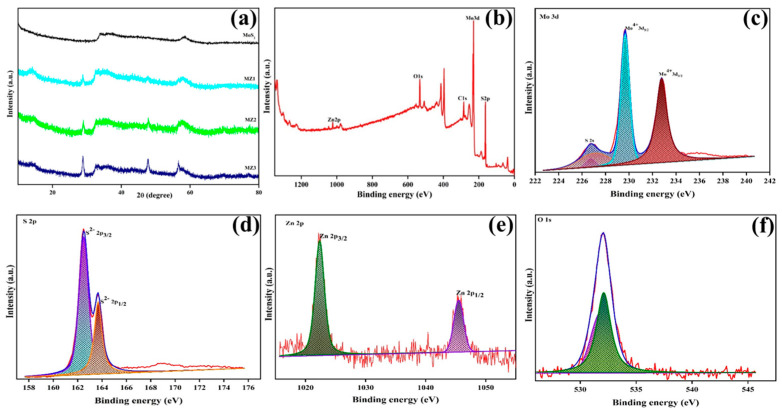
(**a**) XRD pattern of MZ composites. (**b**) XPS full survey spectrum of MZ composite. High-resolution XPS spectra of (**c**) Mo 3d, (**d**) S 2p, (**e**) Zn 2p, and (**f**) O 1s of MZ composite.

Initially, the structural properties of the developed MoS_2_-ZnO composites were investigated through XRD measurements. Figure 2a illustrates the XRD pattern of different concentrations of MZ1, MZ2, and MZ3 nanocomposites. According to the MoS_2_ spectra, the as-prepared materials show mostly an amorphous property. In the composite, the distinct diffraction peaks noticed at 14.1°, 28.7°, 32.3°, and 57.8° may be ascribed to the (002), (004), (100), and (110) reflection planes of MoS_2_, respectively [41,42]. Thus, the XRD spectra successfully indicate the formation of MoS_2_ and it is dominantly present in the composite. In addition, further additional diffraction peaks were observed together with the MoS_2_ materials peak. The minor peaks appeared at 36.2° and 47.7°, confirming the presence of ZnO nanomaterials [43,44] in the composite. In light of this, the XRD analysis suggests the efficient formation of ZnO in MZ nanocomposites. Moreover, the peak intensity of MoS_2_ and ZnO is varied in the MZ composites by varying the ratio between MoS_2_ and ZnO as MZ1, MZ2, and MZ3 in composites. Consequently, the XRD findings indicate the homogeneous formation of ZnO on the MoS_2_ in the MZ nanocomposites.

The XPS characterization technique was used to confirm further the formation of MoS_2_ and ZnO in MZ nanocomposites. XPS analysis delivers information about the chemical nature, electronic state of the elements within the material, and elemental composition of the as-prepared MZ nanocomposite. Figure 2a represents the XPS full-scan spectrum of the MZ2 nanocomposite. As shown in the survey spectrum, Mo, S, Zn, and O elements have been identified for the MZ2 nanocomposite. The deconvolution peaks for the MZ2 nanocomposite are illustrated in Figure 2c–f. In the Mo 3d spectra (Figure 2c), there are two characteristic peaks around 229.65 eV and 232.75 eV for Mo3d_5/2_ and Mo 3d_3/2_, respectively [45]. Notably, the existence of S^2−^ is confirmed by the appearance of S2s peak at 226.7 eV. In addition, the S 2p spectra (Figure 2d) show two prominent peaks at 162.45 eV and 163.67 eV, which may be attributed to the S 2p_3/2_ and S 2p_1/2_. Therefore, the XPS results effectively identify the presence of MoS_2_ as a predominant component in the MZ composite. In addition, two peaks appear for Zn 2p_3/2_ and Zn 2p_1/2_ with binding energies of 1022.29 and 1045.52 eV, respectively, demonstrating the distinctive peaks of Zn^2+^ oxidation state in the MoS_2_-ZnO composite [46,47]. Figure 2f depicts the O 1s spectra with peaks at 532 eV and 531.84 eV. The ZnO lattice oxygen is represented by the peak at 531.6 eV. On the other hand, the peak at 532 eV serves as an illustration of the hydroxyl group produced during surface modification [48]. According to the XRD and XPS analyses results, the successful formation of the MZ composite is evident.

**Figure 3:**
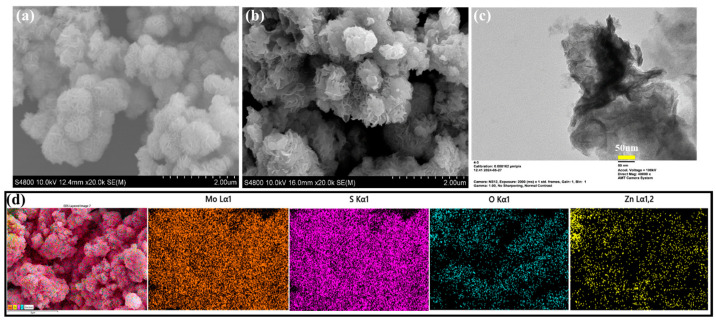
SEM images of (**a**) MoS_2_ and (**b**) MZ2 nanocomposite. (**c**) TEM image MZ2 nanocomposite. (**d**) EDAX elemental mapping of MZ2 composite.

FE-SEM analysis examined the surface morphology of the as-prepared pristine MoS_2_ and MoS_2_/ZnO nanocomposites. Figure 3a depicts an SEM micrograph image of pristine MoS_2_ synthesized using a hydrothermal technique. The pristine MoS_2_ exhibits a surface morphology composed of a large number of nanosheets that are linked via the center. This results in the production of three-dimensional flower-like structures. Furthermore, these nanosheets have a distinct similarity to the petals of flowers. Compared to the as-developed pristine MoS_2_, different surface properties have been observed in the MZ2 and MZ3, as shown in Figure 3b and Figure S1, respectively. It can be observed that the ZnO nanoparticles are anchored on the MoS_2_ petals in the nanocomposite. Moreover, the ZnO is eventually observed throughout MoS_2_. However, with the increase in the concentration of ZnO in the composite (MZ3), an uneven formation and aggregation of ZnO on MoS_2_ petals has been observed, as represented in Figure S1. Thus, the effectual combination of ZnO on MoS_2_ in MZ2 can serve as a more capable candidate for the dye degradation process together with polymers. 

TEM analysis was carried out for the MZ2 nanocomposite to identify further the homogeneity of the nanocomposite (Figure 3c). At this point, the dispersion of ZnO nanoparticles on the MoS_2_ sheet-like structure is clearly observed, which confirms the homogeneity. Furthermore, SEM–EDAX elemental analysis (Figure 3d and Figure S2) of the MZ2 composite was performed to investigate the formation of the composite. As shown in EDAX mapping and spectra results, the presence of Mo, S, Zn, and O elements is found uniformly on the composite, which confirms the excellent formation of ZnO on MoS_2_ and its uniform combination in the MZ composite. The determined weight percentage of the Mo, S, Zn, and O elements in MZ2 is 50.42, 35.22, 5.21, and 12.15%, respectively. According to the microstructural characterization, it can be concluded that the ZnO particles are effectively distributed together with MoS_2_ for forming the homogeneous MZ nanocomposite.

**Figure 4:**
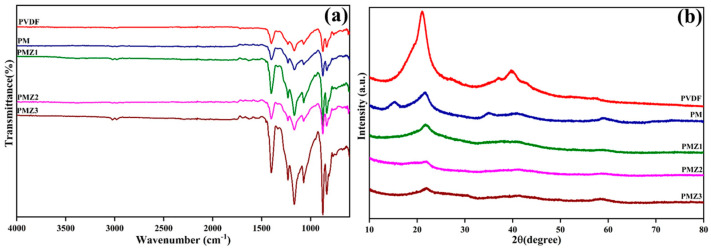
(**a**) FTIR and (**b**) XRD spectra of PVDF, PM, and PMZ composite films.

The as-prepared films were subjected to FT-IR analysis in order to determine the functional groups present in the matrix. Figure 4a displays the FTIR analysis of PVDF, PVDF–MoS_2_, and PMZ composite films. As shown in the PVDF spectra, the asymmetric, symmetric, and wagging stretching of the –CH_2_ group is exhibited by peaks at 3027, 2979, and 1398 cm^−1^, respectively. The peaks at 1226, 1162, and 1070 cm^−1^ are attributed to the stretching vibration of the –CF_2_ group. The peaks at 832 and 870 cm^−1^ represent C-F bending and C-H wagging [49,50,51,52,53]. The observed peaks are effectively matched with PVDF material. The FTIR analysis was performed on the binary (PM) and ternary (PMZ) hybrid films to further confirm the excellent integration of nanomaterials into the polymer matrix. The spectra of PM and different ratios of PMZ films show that the most dominant peaks are related to the PVDF material. This phenomenon occurs because of the most significant amount of PVDF in the film compared to other compounds. It was noted that the usual MoS_2_ and ZnO peaks had been masked by other pre-existing peaks of PVDF. However, a slight existence of –OH, Mo=S, S-S bond, and Zn-O groups has been observed in their corresponding regions [54,55,56]. The FT-IR analysis confirms the presence of functional properties in the polymer and the successful fabrication of the composite films.

Figure 4b illustrates the XRD patterns of pristine PVDF, PM, and PMZ (PMZ1, PMZ2, and PMZ3) composite films. For the PVDF film, the distinct diffraction peaks at 19.43°, 20.9°, 26.88°, 36.97°, and 38.85° can be attributed to the lattice planes of α (110), β (110), α (021), β (201), and γ (211) planes, respectively [57,58,59,60,61]. In this, the most dominant peaks correspond to the β phase of the PVDF. In most cases, the β phase of the PVDF formation has been influenced by the solvents during the film preparation steps. Compared to the PVDF-only film, a different peak observation and changes in peak intensity of PVDF have been observed after the inclusion of inorganic materials into the polymer matrix. The XRD patterns of the PM composite film exhibit a widening and decreasing reduction in the intensities of the diffraction peaks, which indicates that certain α and γ phases probably transitioned into the β phase [62]. Furthermore, similar phase shifts have been seen in the PMZ composite films, which may be caused by the addition of inorganic materials effectually included in PVDF. In the case of the binary PM film, additional peaks are exhibited together with PVDF peaks. The observed distinct diffraction peaks at nearly 14.5° and 34.1° correspond to the MoS_2_ [38,63]. Moreover, a very minimal diffraction peak was observed in the ternary PMZ film for ZnO [44] along with PVDF and MoS_2_. XRD results of the films indicate the efficient inclusion of MZ in the hybrid film. 

**Figure 5:**
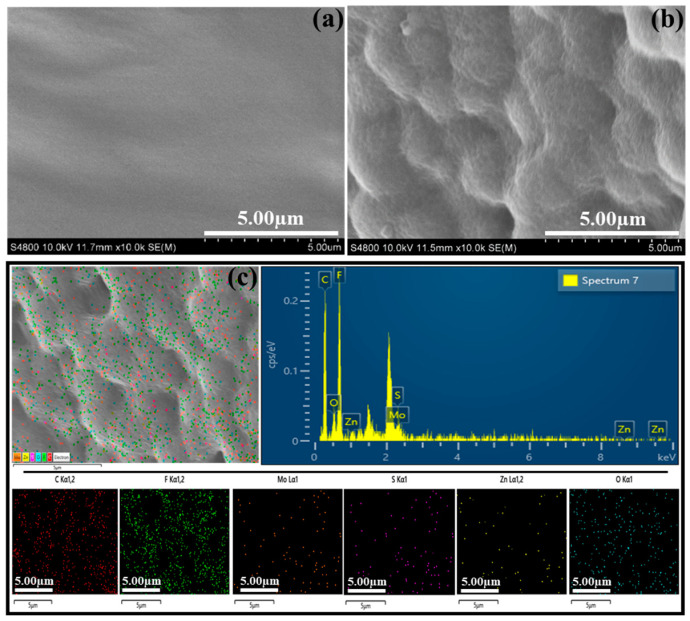
FE-SEM images of (**a**) PVDF and (**b**) PMZ2 composite films. (**c**) EDAX spectrum and elemental mapping of PMZ composite film.

The surface morphology of the as-prepared PVDF and PMZ films were characterized through SEM analysis, and the corresponding micrograph images are represented in Figure 5a,b, respectively. In the case of the PVDF film (Figure 5a), a nearly smooth and dense surface morphology has been obtained. Different morphological characteristics have been observed after the inclusion of MZ2 into the polymer matrix as a hybrid film (Figure 5b). In the PMZ film, an irregular and non-smooth surface has been observed due to the excellently included ZnO, containing a flower-like structure of MoS_2_ microspheres in the overall film. Thus, the presence of inorganic composite microspheres in the polymer prohibits the dense film formation and leads to an uneven and slight porosity-like nature on the surface of the composite film. Compared to the dense film, the uneven wrinkle-like surface morphology with porosity can provide certain benefits, such as an increase in the active surface region and excellent movement of water molecules with certain chemical compounds. The possible penetration of water molecules together with contaminates inside of the PVDF-based film can led to higher degradation of contaminates during the sonocatalytic organic pollutant degradation from water. 

To further demonstrate the uniformity of films, EDAX findings were performed on the PVDF and PMZ films. The EDAX mapping and spectra results are illustrated in Figure 5c and Figure S3 for PVDF and PMZ films, respectively. For the PVDF film, carbon (C) and fluorine (F) elements were observed, which were obviously from the PVDF. In the case of the PMZ film (Figure 5c), together with C and F elements, additional elements of molybdenum (Mo), sulfur (S), zinc (Zn), and oxygen (O) have been detected. The observed weight percentages of F, C, Mo, S, Zn, and O elements in PMZ are 38.87, 55.86, 2.29, 1.31, 0.03, and 1.64%, respectively. These hybrid film EDAX results successfully confirm the efficient and uniform distribution of inorganic nanomaterials in the PVDF polymer film. 

**Figure 6:**
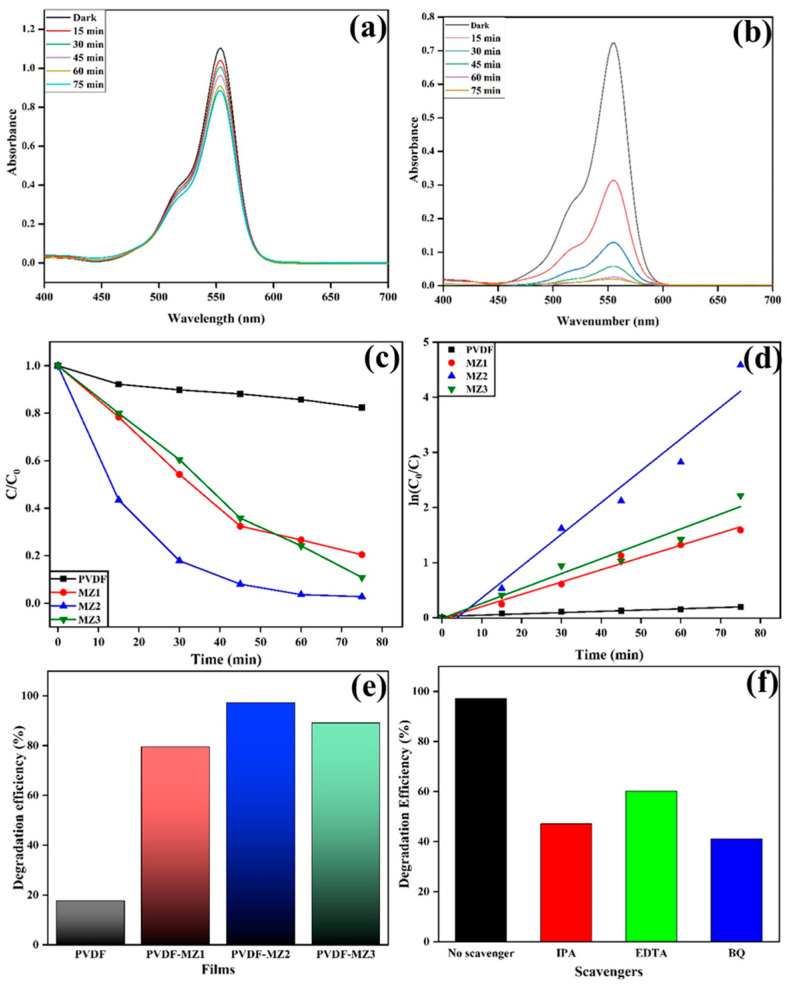
UV–vis absorbance spectra for RhB dye degradation by (**a**) PVDF and (**b**) PMZ2 films; (**c**,**d**) sonodegradation pseudo-first-order kinetic models of PVDF and PMZ films. (**e**) Sonocatalytic degradation efficiency of PVDF and PMZ composite films. (**f**) Effect of various scavengers on degradation efficiency by PMZ2 film.

The sonocatalytic efficacy of PVDF, PM, PMZ1, PMZ2, and PMZ3 nanocomposite films has been assessed by examining the degradation of RhB dye. At first, the adsorption–desorption equilibrium for sonocatalytic dye degradation was performed by placing the film in the RhB-containing solution in the dark with stirring for 30 min. No significant changes have been seen in the aqueous solution, and further measurements of UV–visible absorbance have been recorded. Afterward, the sonocatalytic degradation experiment was performed by keeping the aqueous dye solution with film in a sonication bath for 75 min. At regular time intervals (15 min), 4 mL of aliquots was collected, and the absorbance was measured through UV–vis spectroscopy. Figure 6a,b illustrate the UV–vis absorbance spectra of RhB aqueous solution with the PVDF and PMZ2 films. It is discovered that the strength of the absorption band in the PMZ2 film declines with increasing ultrasonic irradiation duration and diminishes after 75 min. In the case of the PVDF film, small changes in the absorption band have been observed after the reaction. According to this observation, the PMZ2 film has a greater capacity for degrading RhB compared to the PVDF and other composite films. The RhB degrades sonocatalytically according to the pseudo-first-order model, which is derived from the following equation:

ln (*C*_0_*/C*) = *kt* (2)

where *C_0_* and *C* indicate the RhB solution concentration at initial and particular time intervals (*t*), and *k* indicates the first-order rate constant (min^−1^). In this case, the rate constant was calculated based on the gradient of the regression line. Consequently, it was discovered that the degradation rate constants for PVDF, PMZ1, PMZ2, and PMZ3 were, respectively, 0.0023 min^−1^, 0.02228 min^−1^, 0.05772 min^−1^, and 0.02705 min^−1^ (Figure 6c,d). Furthermore, when subjected to ultrasonic irradiation, the degradation efficiency of RhB was found to be 17.7% (R^2^ = 0.94), 79.83% (R^2^ = 0.985), 97.23% (R^2^ = 0.96), and 89.13% (R^2^ = 0.956) for the PVDF film, and the PMZ1, PMZ2, and PMZ3 films, respectively (Figure 6e). Based on the findings, it is notable that the PMZ2 film demonstrates superior sonocatalytic performance compared to the PVDF and other composite films. 

**Figure 7:**
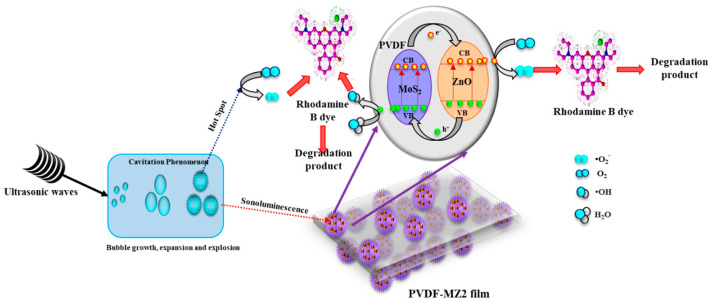
Probable sonocatalytic degradation mechanism of RhB by PMZ film using ultrasound irradiation.

The probable sonocatalytic degradation mechanism of RhB by the PMZ2 film is schematically illustrated in Figure 7. The ultrasound irradiation on RhB aqueous solution generated the acoustic cavitation phenomenon in which the cavitation bubbles develop, expand, and suddenly collapse, resulting in the generation of sonoluminescence and hot spots [15,64,65]. Through pyrolysis, these hotspots accelerate the disruption of H_2_O molecules to generate hydroxyl (•OH) radicals for degrading the dye pollutants [66]. Consequently, the sonoluminescence effect generated lights that excited the MZ2 in the PMZ2 film and acted as a sonocatalyst for degrading RhB dye [67]. The light generated excites both MoS_2_ and ZnO to produce sonogenerated electrons and holes. The excited e^−^ from CB of MoS_2_ can be transferred into CB of ZnO. Simultaneously, sonogenerated holes from VB of ZnO move to VB of MoS_2_. This movement will reduce the electron–hole recombination and increase the sonocatalytic degradation performances. These sonogenerated electrons and holes move toward the surface of the film. These electrons reduce the dissolved O_2_ molecules in an aqueous solution and produce superoxide radical (•O_2_^−^). Meanwhile, the generated holes oxide the H_2_O molecule and generate hydroxyl (•OH) radicals [68]. These free radicals are then involved in dye degradation by oxidizing the dye molecules and generating CO_2_, H_2_O, etc. The sonocatalytic dye degradation mechanism is expressed as follows:

US (Hot spot) + H_2_O → •OH + •H (3)

US (Sonoluminescence) + sonocatalyst → e^−^ + h^+^ (4)

e^−^ + O_2_ → •O_2_^−^ (5)

h^+^ + H_2_O → •H + •OH (6)

•O_2_^−^ + Rh B dye → degradation products (7)

•OH + Rh B dye → degradation products(8)

Furthermore, the nanocomposite materials (MZ) and PVDF exhibit improved piezoelectric properties due to the mechanical stress induced during ultrasonic irradiation. Consequently, the MZ nanocomposite spontaneously polarizes when subjected to prolonged ultrasonic irradiation, resulting in the generation of electron–hole pairs [38]. Additionally, the piezoelectric polarization can generate an internal electric field as a result of the pressure developed during ultrasonic treatment. The presence of the inherent electric field facilitates the segregation of electron–hole pairs and also hinders the recombination of produced electron–hole pairs [69]. The electron–hole pairs migrate towards the surface of the film and subsequently produce reactive oxygen species such as •O_2_^−^, •OH, etc., by their interacting with dissolved O_2_ and H_2_O molecules in an aqueous solution. Thus, these ROS are also implicated in the breakdown of RhB dye. Additionally, the homogeneous distribution of MZ nanocomposites in the PVDF polymer matrix enhances the surface area, hence improving sonocatalytic activity. Meanwhile, PVDF acts as a structural framework that provides mechanical resilience and rigidity, protecting against breaking or distortion under mechanical stress during ultrasound vibration [70]. 

**Figure 8:**
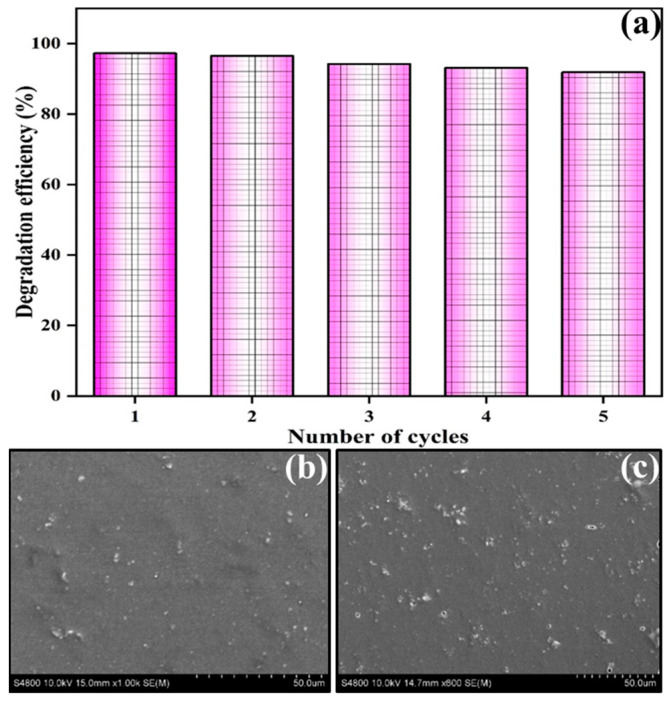
(**a**) Cyclic stability and performance results of PVDF–MZ2 film. SEM images of PMZ2 film (**b**) before and (**c**) after the sonocatalytic degradation process.

The trapping experiment was performed to identify the active species involved in the sonocatalytic degradation of RhB dye by the PMZ2 film, as represented in Figure 6f. Scavengers such as isopropanol (IPA), p-benzoquinone (BQ), and ethylenediamine tetraacetic acid (EDTA) was used to capture •OH, •O_2_^−^, and h^+^, respectively. Sonocatalytic degradation efficiencies of 47.12%, 60.12%, and 41.14% has been observed for scavengers such as IPA, EDTA, and BQ, respectively. As a result, the degradation efficiency was found to decrease using BQ and IPA, indicating •O_2_^−^ and •OH active species are most responsible for RhB degradation using PMZ2 film. The stability and reusability performance of the PMZ2 film was demonstrated through cyclic degradation of RhB dye under ultrasonic irradiation. After finishing each cycle, the film was removed, washed, oven-dried, and reused for the next cycle, while the other reaction conditions were maintained. As observed in Figure 8a, the PMZ2 film exhibited reasonable sonocatalytic activity after five rounds of RhB degradation. It has been observed that after 75 min of each cycle, the sonocatalytic degradation percentage for RhB was comparable. The modest variation in dye degradation was attributed to the loss of the material polarizing capacity during the process of reusing under continuous ultrasonic stress. After cyclic performance, the film surface was characterized through SEM analysis. Figure 8b,c illustrate the surface morphology of the PMZ2 film before and after the RhB dye degradation process. It shows that the film surface does not exhibit any significant changes before and after the cyclic performance, which indicates that the PMZ2 film has efficient stability during ultrasonic processes for dye degradation. The PMZ film has been determined to provide several advantages in terms of environmental pollutant degradation.

## 4. Conclusions

In this work, a PVDF polymer was combined with different ratios of hydrothermally generated MoS_2_-ZnO (MZ) nanocomposites to effectively create a PVDF–MoS_2_-ZnO (PMZ) hybrid nanocomposite film. The developed PMZ2 hybrid film showed impressive sonocatalytic degrading effectiveness on RhB dye when exposed to ultrasonic mechanical vibration, as compared to pristine PVDF and other PMZ films. The determined degradation efficiency of RhB using PVDF, PMZ1, PMZ2, and PMZ3 film was found to be 17.7%, 79.83%, 97.23%, and 89.13%, respectively. The enhanced efficacy was achieved by the presence of MZ nanocomposites in the PVDF, which increase the piezocatalytic characteristics of PVDF while also reducing electron–hole pair recombination and increasing catalytic sites for dye degradation in the PMZ film. The generation of •O_2_^−^ and •OH reactive oxygen species, which has been identified through scavenging studies, is responsible for the degradation of RhB by the PMZ2 film under sonocatalytic conditions. Furthermore, the hybrid PMZ nanocomposite films exhibited excellent stability and reusability, evident from their steady cyclic degradation performances after five cycles of ultrasonic vibration without any noticeable changes in their structure and degrading efficiency. This study proposes a promising approach to create and produce a durable hybrid catalytic nanocomposite film that may efficiently break down contaminants in wastewater, thereby mitigating environmental issues.

## Data Availability

The original contributions presented in the study are included in the article/Supplementary Materials, further inquiries can be directed to the corresponding author.

## Supplementary Materials

The following supporting information can be downloaded at: https://www.mdpi.com/article/10.3390/polym16152213/s1, Figure S1. SEM micrograph images of MZ3 nanocomposite. Figure S2. EDAX spectra of MZ2 nanocomposite. Figure S3. EDAX mapping and spectra of PVDF film. Table S1. Comparative performance results of PVDF based membranes/films. References [37,70,71,72,73,74,75,76] are cited in the Supplementary Materials.
